# The Complexity of Diphtheria Toxin

**Published:** 1914-04-25

**Authors:** 


### The Complexity of Diphtheria Toxin.

Some important researches from the Wellcome Laboratories on the toxin of diphtheria and on the constitution of the antitoxins which neutralise it are published in the *Journal of Hygiene.* Mr. Glenny was investigating the deterioration of diphtheria antitoxins which have been kept for a long time, and was thereby led to the following conclusions. "The constituent of diphtheria toxin which is acutely lethal in its action is not identical with that which causes the local reaction at the seat of injection." (This local reaction is exhibited as necrosis of the skin in experimental cases, and possibly compares with the local tonsillar necrosis seen clinically.) Also that "the power of a serum to neutralise the acutely lethal constituent of a toxin may vary independently of its power to neutralise the constituent causing local reaction." It was long ago suggested that both the diphtheria toxin and the antitoxin may be complex in character; these researches, which are being continued, seem likely to confirm that idea, and also possibly to throw important light on the constituent bodies. Such knowledge might quite conceivably be of great clinical utility.

